# Tropical Plant–Herbivore Networks: Reconstructing Species Interactions Using DNA Barcodes

**DOI:** 10.1371/journal.pone.0052967

**Published:** 2013-01-08

**Authors:** Carlos García-Robledo, David L. Erickson, Charles L. Staines, Terry L. Erwin, W. John Kress

**Affiliations:** 1 Department of Botany, National Museum of Natural History, Smithsonian Institution, Washington, D.C., United States of America; 2 Department of Entomology, National Museum of Natural History, Smithsonian Institution, Washington, D.C., United States of America; Centro de Investigación y de Estudios Avanzados, Mexico

## Abstract

Plants and their associated insect herbivores, represent more than 50% of all known species on earth. The first step in understanding the mechanisms generating and maintaining this important component of biodiversity is to identify plant-herbivore associations. In this study we determined insect-host plant associations for an entire guild of insect herbivores using plant DNA extracted from insect gut contents. Over two years, in a tropical rain forest in Costa Rica (La Selva Biological Station), we recorded the full diet breadth of rolled-leaf beetles, a group of herbivores that feed on plants in the order Zingiberales. Field observations were used to determine the accuracy of diet identifications using a three-locus DNA barcode (*rbcL*, *trnH-psbA* and ITS2). Using extraction techniques for ancient DNA, we obtained high-quality sequences for two of these loci from gut contents (*rbcL* and ITS2). Sequences were then compared to a comprehensive DNA barcode library of the Zingiberales. The *rbcL* locus identified host plants to family (success/sequence = 58.8%) and genus (success/sequence = 47%). For all Zingiberales except Heliconiaceae, ITS2 successfully identified host plants to genus (success/sequence = 67.1%) and species (success/sequence = 61.6%). Kindt’s sampling estimates suggest that by collecting ca. four individuals representing each plant-herbivore interaction, 99% of all host associations included in this study can be identified to genus. For plants that amplified ITS2, 99% of the hosts can be identified to species after collecting at least four individuals representing each interaction. Our study demonstrates that host plant identifications at the species-level using DNA barcodes are feasible, cost-effective, and reliable, and that reconstructing plant-herbivore networks with these methods will become the standard for a detailed understanding of these interactions.

## Introduction

Plants, together with their associated insect herbivores, represent more than 50% of all known species on earth [1]. This overwhelming biological diversity is in part the product of the coevolutionary processes that have taken place between these two ecological partners [1]–[4]. Leaf consumption by phytophagous insects also represents one of the major conduits of energy as it flows through the food chain to various trophic levels [1]. The first step in understanding how these ecological and evolutionary interactions create and maintain biological diversity is to accurately determine the intricate associations and networks between insect herbivores and their host plants.

Developing and implementing cost effective and reliable methods to identify insect herbivore diets has been a challenge. The most common strategies have included: 1) direct observations of herbivory in the field; 2) laboratory feeding trials [5]; 3) morphological analyses of gut contents; and 4) stable isotope techniques [6], [7]. Unfortunately, identifying the diet of a whole community of insect herbivores using these methods is challenging. Field observations are problematic in habitats that are difficult to access, such as the forest canopy or underground. Direct observations are also greatly limited by the ability of the researcher to correctly identify the species involved in the interactions [8]. In laboratory feeding trials, insects will often feed on host plants not normally consumed in nature, and thereby the diet breadths of the insects can be overestimated [5]. For morphological and stable isotope analyses the resolution of the taxonomic and/or ecological information is often very limited.

**Figure 1:**
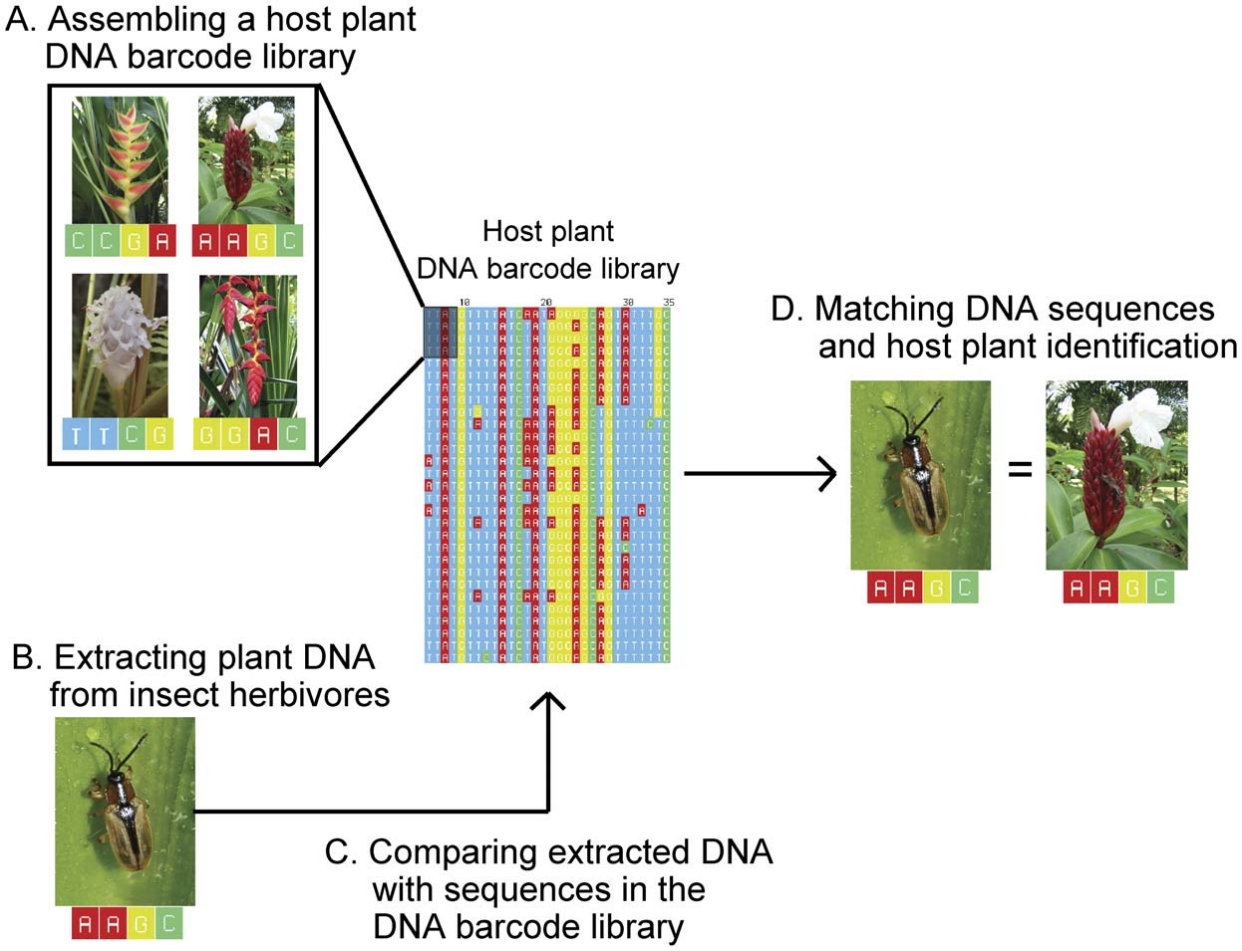
Diet identification using DNA barcodes. First we assembled a DNA barcode library containing sequences of all potential host plants (*A*). After collecting insect herbivores in the field (*B*), we extracted plant DNA from gut contents (*C*). Host plant identifications were performed by comparing DNA sequences extracted from insect herbivores to sequences in the DNA barcode library using the BLAST algorithm and default search parameters (*D*).

An alternative to identify insect diets is to use plant DNA sequences extracted from the guts of insects (Figure 1). This method is especially effective when DNA sequences of gut contents can be compared to plant DNA sequences already known and assembled into reference libraries (Figure 1). Previous studies have demonstrated that molecular markers have the potential to identify insect herbivore diets at the taxonomic level of family and genus [9], [10]. However, the unambiguous identification of host plants at the species-level and for whole communities of insect herbivores using molecular markers still remains unproven. Such species-level identification studies will require a full DNA reference library of host plant species in the target community, improved DNA extraction techniques from partially degraded DNA found in insect guts, and multiple molecular marker to increase discriminatory power [9], [10] (Figure 1).

To assess the efficacy and accuracy of using DNA barcodes as molecular markers to determine host species in whole communities of insect herbivores, we performed a field and laboratory experiment on a well-studied plant-insect interaction: plant hosts in the order Zingiberales and their rolled-leaf beetle herbivores in the genera *Cephaloleia* and *Chelobasis* (Cassidinae: Chrysomelidae). We performed this study at La Selva Biological Station (hereafter La Selva) in a tropical rain forest habitat in Costa Rica, Central America [11]. This guild of herbivores is known as the “rolled-leaf” beetles because their life cycle occurs inside the young rolled leaves of their primarily Zingiberalian host plants [12]. The taxonomy and classification of both the Zingiberales and the rolled-leaf beetles have been thoroughly studied at La Selva during the last 40 years [13].

This well-studied plant-herbivore system allowed us to determine the ability of three plant DNA barcode loci (*i.e., rbcL*, ITS2 and *trnH-psbA*) to accurately identify insect diets [14], [15]. We first recorded in the field using direct observations over the course of two years the complete diets of each species of rolled-leaf beetle at La Selva. We then generated a complete DNA barcode library for all of the species of Zingiberales at La Selva. Finally, we resampled in the field multiple individuals of each species of beetle, preserved them in liquid nitrogen, and sequenced the gut contents of each beetle using standard methods of Sanger sequencing. We compared the resultant DNA sequences with our previously generated reference DNA barcode library to identify the beetle diets. The results of the DNA barcode analyses were then compared to our direct field observations of herbivore-plant host interactions. Identifications to the species-level were possible by the development of a new DNA extraction protocol and genetic markers that are applicable to the identification of diets in many plant-herbivore systems (*see* Methods section). Finally, we estimated the sampling effort required to identify all plant-herbivore interactions in a community of rolled-leaf beetles using DNA barcodes.

## Results and Discussion

### A Comprehensive Field Survey of Rolled-leaf Beetle – Zingiberales Host Plant Interactions

**Figure 2:**
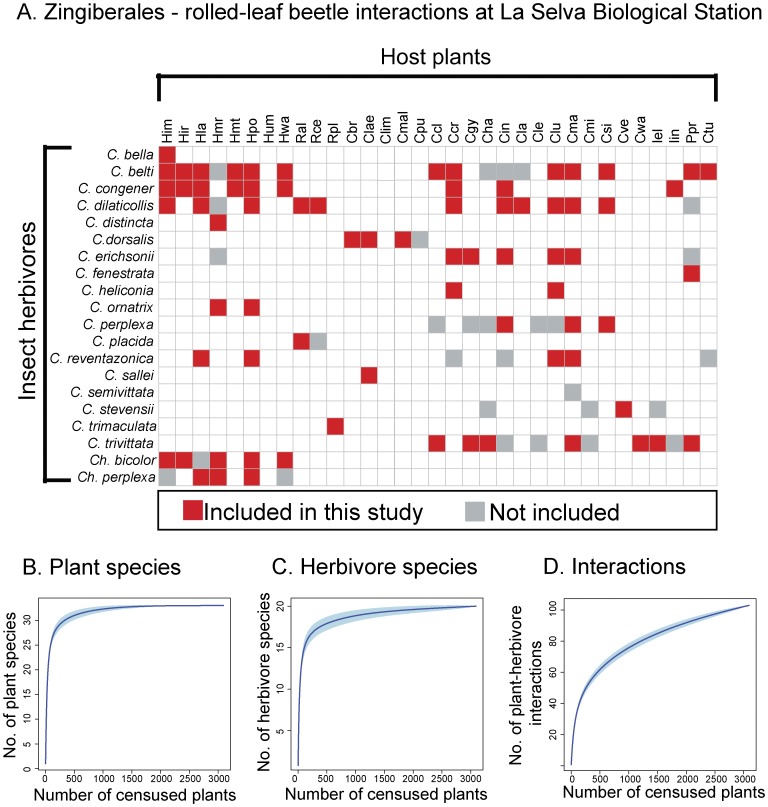
A comprehensive survey of rolled-leaf beetles and their associations with Zingiberales at La Selva Biological Station, Costa Rica. *A*. Interactions recorded for two years during rainy and dry seasons (October 2008 - February 2009 and October 2010– February 2011). Each matrix element represents an association between an insect herbivore species (rows) and a host plant species (columns). Interactions in red were recorded during the second year of this study and selected for diet analyses. *B*. Sampling effort and accumulation curves of plant and insect herbivore species and their interactions at La Selva (Mean ± SD). Total number of host plants censused = 3092. Total number of insect herbivore records = 7359. Host plant species abbreviations: Heliconiaceae. Him = *Heliconia imbricata*. Hir = *H. irrasa*. Hla = *H. latispatha*. Hmr = *H. mariae*. Hmt = *H. mathiasiae*. Hpo = *H. pogonantha*. Hum = *H. umbrophila.* Hwa = *H. wagneriana*. Zingiberaceae. Ral = *Renealmia alpinia*. Rce = *R. cernua*. Rpl = *R. pluriplicata*. Costaceae. Cbr = *Costus bracteatus*. Clae = *C. laevis*. Clim = *C. lima*. Cmal = *C. malortieanus*. Cpu = *C. pulverulentus.* Marantaceae. Ccl = *C. cleistantha*. Ccr = *C. crotalifera*. Cgy = *C. gymnocarpa*. Cha = *C. hammelii*. Cin = *C. inocephala*. Clas = *C. lasiostachya.* Cle = *C. leucostachys*. Clu = *C. lutea*. Cma = *C. marantifolia*. Csi = *C. similis*. Cve = *C. venusta*. Cwa = *C. warscewiczii*. Iel = *Ischnosiphon elegans*. Iin = *I. inflatus*. Ppr = *Pleiostachya pruinosa*. Cannaceae: Ctu = *Canna tuerckheimii*.

Over the two years of our field study, we recorded by direct observations at La Selva 20 species of rolled-leaf beetles, 33 species of Zingiberales, and 103 plant-herbivore interactions (Figure 2*A*). Accumulation curves show that our efforts to record the diets of 7359 individual rolled-leaf beetles on 3092 host plants resulted in a comprehensive account of species and their interactions for this plant-herbivore system (Figure 2*B*). This survey was essential in order to determine the fraction of the total network recovered as well as any identification errors from using DNA barcodes.

### DNA Barcode Libraries of Host Plants

Previous studies determining animal diets using molecular markers compared diet sequences to public DNA databases such as GenBank [9], [10]. In these cases, host plant identifications were successful to tribal and family taxonomic levels [9]. However, identifications to genus - or species-levels were frequently ambiguous [10]. The use of public DNA databases is limited for such studies because they do not always include all potential host plants, and, therefore, diet identifications may be incorrect [10].

Incorrect identifications can be reduced by removing from the DNA library those species that have never been recorded in the study area [9]. However, this approach has serious limitations, especially for highly diverse ecosystems such as tropical forests, where species inventories are incomplete. Misidentification rates can also be reduced using conservative parameters in Bayesian reconstruction of phylogenetic relationships [9]. Although conservative parameters can reduce error rates, they also greatly reduce the taxonomic resolution of diet identifications [9]. Previous studies have concluded that the best strategy to increase the accuracy and taxonomic resolution of diet identifications using DNA barcodes is to have a complete DNA barcode reference library of host plants [9], [10].

We successfully generated *rbcL* and ITS2 libraries for most Zingiberales at La Selva (Figure 2*A*). It was not possible to amplify high quality *trnH-psbA* sequences for most of the Zingiberales so this locus was not informative for this study. In addition, the ITS2 locus could not be amplified for plants in the family Heliconiaceae. However, ITS2 sequences were successfully amplified for all the remaining plants in the families Cannaceae, Costaceae, Marantaceae, and Zingiberaceae.

### Identification of Insect Herbivore Diets Using Molecular Markers

To determine the reliability of host plant identifications using DNA barcodes, we sampled individuals representing all interactions among Zingiberales and their rolled-leaf beetle herbivores recorded during the second year of this study (N_individuals_ = 244, N_interactions_ = 74, Figure 2*A*). These interactions represent 72% of all Zingiberales – rolled-leaf beetle interactions at La Selva (Figure 2*A*).

Plant DNA recovery rates from rolled-leaf beetle gut contents ranged between 45.9% and 48.7% (Table 1). The *trnH-psbA* locus was rarely amplified from gut content samples and the resulting sequences were of poor quality. Thus, we based our results on *rbcL* and ITS2 only.

**Table 1:** Percent of success extracting plant DNA from gut contents and identification success of the resulting DNA sequences for the DNA barcodes *rbcL* and ITS2.

			IDENTIFICATION SUCCESS PER SEQUENCE (%)			
DNA barcodes	Sample size	DNA extraction success per collection (%)	Family	Genus	Species	Species misidentifications (%)
*rbcL*	244	48.7	58.8	47.0	–	0
ITS2	159	45.9	–	67.1	61.6	0.6

**Figure 3:**
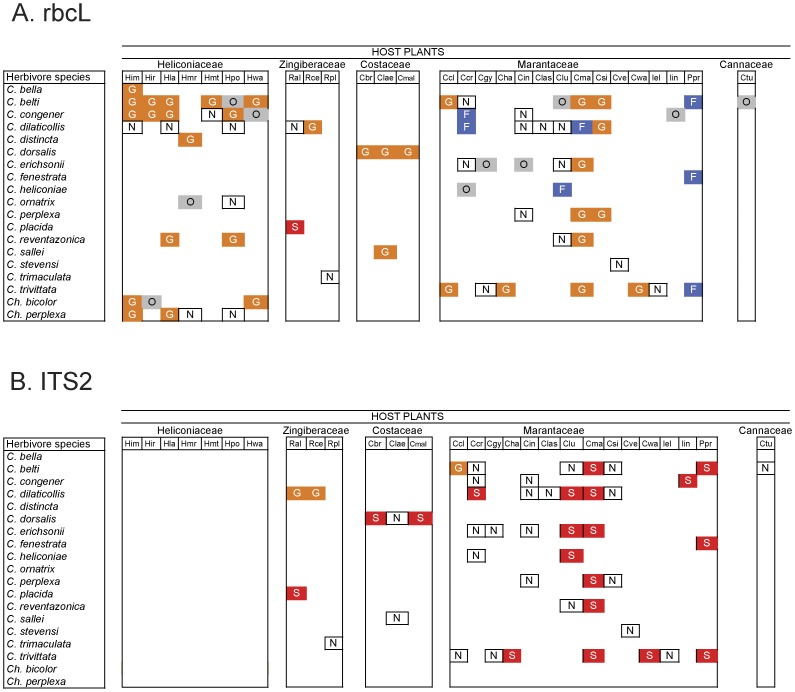
Identification of insect-host plant associations using DNA extracted from rolled-leaf beetle gut contents. Diet identification was performed using the molecular markers: *A*. *rbcL* and *B*. ITS2. Each matrix element represents the associations between a rolled-leaf beetle species (rows) and a host plant (columns). N = interaction failed to be identified by the molecular marker. Taxonomic resolution of successful host plant identifications: O = Order, F = Family, G = Genus, S = Species. Total of sequences = 403 (DNA sequences extracted from insect gut contents are included in Supplement S1). Plant species abbreviations as in Figure 2.

The *rbcL* sequences recovered from insects, when compared with the DNA barcode library, indicated that 47% to 58.8% of the sequences were successfully identified to plant family and genus, respectively (Figure 3*A*, Table 1). Although the ITS2 DNA barcode marker failed to identify host plants in the family Heliconiaceae (Figure 3*B*), this gene region had a high rate of success in the identification of hosts to genus (61.6%) and species taxonomic levels in other Zingiberales (67.1%) (Figure 3*B*, Table 1). The remaining DNA sequences were of poor quality and did not match any sequence in the DNA library.

It is important to note that when we compared the poor quality *rbcL* and ITS2 sequences to the GenBank database, they were assigned to incorrect hosts (Supplement S1). These spurious identifications show the importance of generating local DNA barcode libraries to accurately identify insect diets using these molecular markers.

Our results show that each of these three plant DNA barcode loci is not as universal as desired in such studies and that more than one locus must be used when reconstructing a network of plant-herbivore interactions. For example, long A-T repeats in the *trnH-psbA* region of species of the Zingiberales seem to preclude amplification during the polymerase chain reaction [16] **.** The DNA barcode ITS2 could be successfully amplified for samples of Heliconiaceae. However, the sequences obtained were not useful for plant identification, as the ITS2 region of members of the Heliconiaceae apparently has multiple small copies [17]. These results show the importance of validating molecular methods, as their accuracy will vary among molecular markers and study systems. When using DNA barcodes to identify insect diets, it is important to determine for each molecular marker the optimum taxonomic resolution and taxonomic coverage for particular host plants (*i.e.,* the limitations of each marker to identify different taxa included in the study). A potentially useful marker to identify insect diets is the trnL-intron UAA (P6 loop). This short molecular marker (<100 bp) can be easily amplified from degraded DNA samples such as feces. However, its taxonomic resolution is limited and should be used in studies aimed at determining insect diets to the plant family taxonomic levels only [18].

All *rbcL* sequences were assigned to the correct plant family or genus (Table 1). When using ITS2, three samples did not match the host where the beetles were collected. Two samples of *C. perplexa* matched a host plant used by this species at higher elevation (*Cephaloleia perplexa*, host plant: *Calathea similis* (Marantaceae), diet identified as *C. lasiostachia* (Marantaceae), sequences 112858035, 112858396, Supplement S1). These records most likely represent previous feeding events.

Only one sample of *Cephaloleia placida,* a specialist that feeds on plants in the genus *Renealmia* (Zingiberaceae) was assigned to an incorrect host, *Calathea lutea* (Marantaceae; *Cephaloleia placida,* sequence 112858075; Supplement S1). DNA contamination can be a serious issue with PCR using general plant primers [19]. The most likely cause of this misidentification is DNA contamination as all other samples of *C. placida* were successfully identified to the correct plant species (Figure 3*B*, Supplement S1).

**Figure 4:**
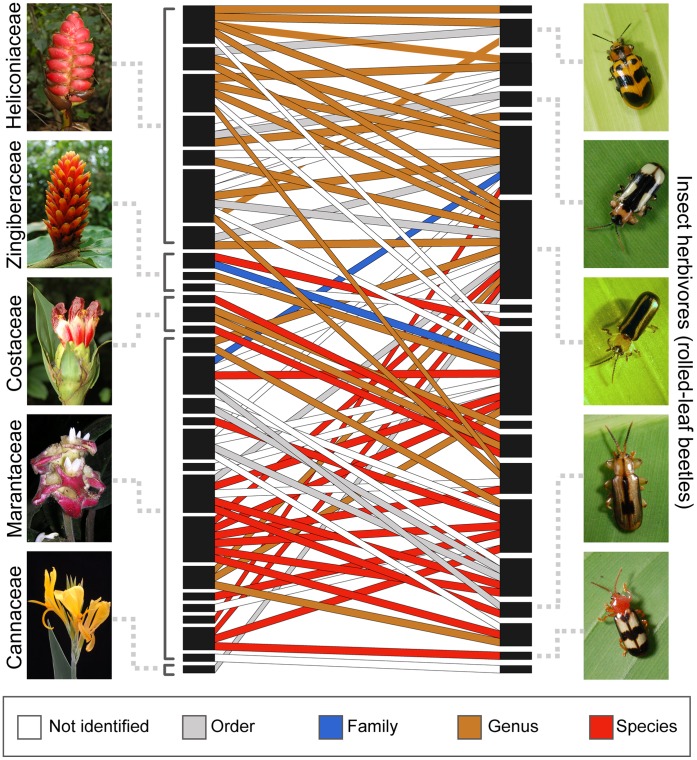
Reconstruction of a tropical plant-herbivore network using DNA extracted from insect gut contents. Rectangles represent insect herbivore and host plant species. Lines connect insect herbivores to their host plants. Line colors represent the taxonomic resolution at which each host plant association was identified. Rectangle size is proportional to its number of interactions. Host plant associations were inferred from *rbcL* and ITS2 DNA fragments. Fragments were compared to host plant DNA barcode libraries containing sequences of all potential hosts in the study area. Total of insect species = 19. Total of plant species = 28. Total of interaction = 74. (*see* matrix of associations in Table S1).

When combining the probabilities of successfully extracting plant DNA from gut contents (45.9–48.7%) and the probability of identification of *rbcL* and ITS2 sequences (Table 1), we successfully identified host plant associations to order (73%), families (61%), genera (59%), and species (26%) (Figure 4, *see* matrix of associations in Table S1). Our success identifying host plants to genus is higher than in other studies as we used more than one molecular marker (51–56% success of identification to genus in references [9]–[10]). A novel feature of this study is our high success unambiguously identifying closely related host plant species (61.6% of amplified sequences, Table 1). This study demonstrates that if full DNA barcode libraries of potential host plants are compiled, it is possible to successfully reconstruct a plant-herbivore network of interactions at the species level.

### Field Sampling Effort and Host Plant Identification Using DNA Barcodes

**Figure 5:**
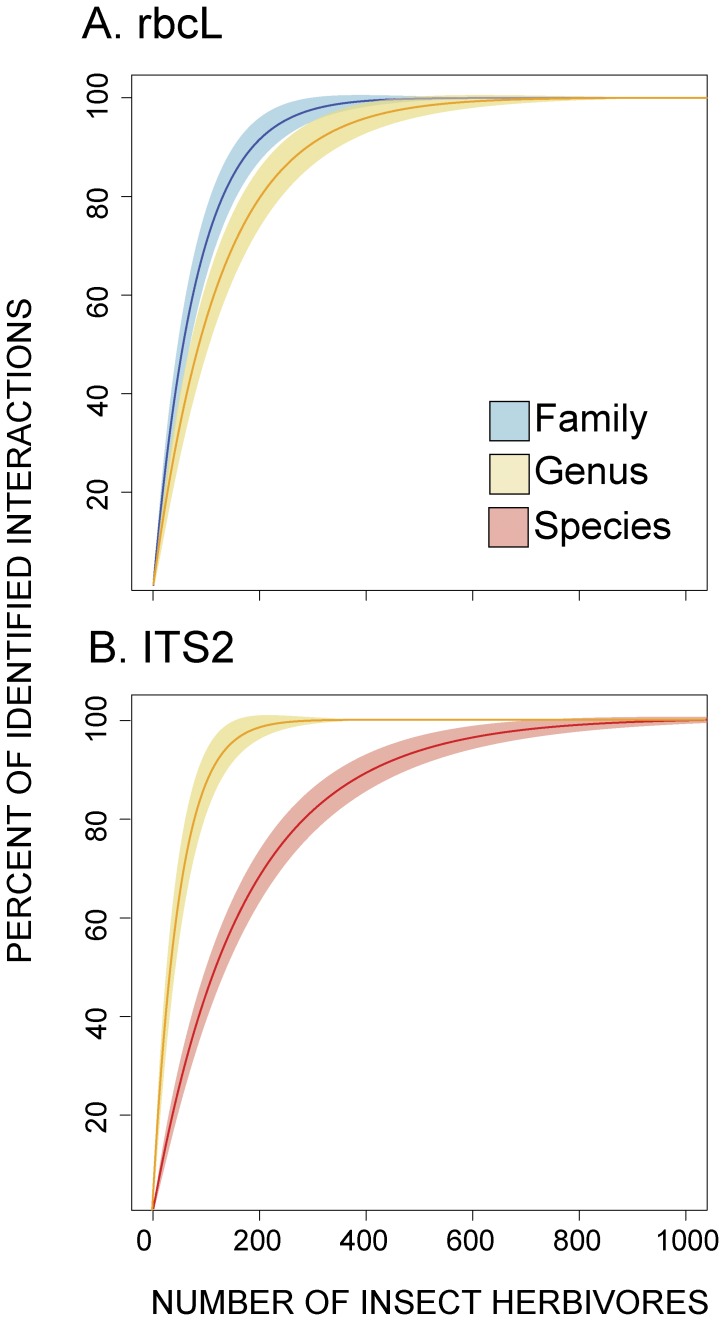
Sampling effort and successful identification of insect herbivore host plants using two molecular markers (Mean ± SD). Diet identifications were performed by comparing DNA sequences from insect gut contents with DNA barcode libraries containing sequences of all potential host plants present in the study area (identification probabilities used to estimate sampling efforts are included in Table 1). *A*. *rbcL* DNA fragments allowed the identification of host plants to the family and genus taxonomic levels. *B*. ITS2 sequences identified insect hosts to the genus and species taxonomic levels.

Using Kindt’s exact accumulators (Equations 1–3), we estimated the sample size required to identify to the highest possible taxonomic resolution all the interactions included in this study (Figure 5). Using the *rbcL* barcode, it was possible to identify 99% of the host plants to family by collecting 500 rolled-leaf beetles. In addition, 99% of the interactions can be identified to genus by collecting ca. 700 rolled-leaf beetles (Figure 5*A*). The ITS2 barcode successfully identified to genus and species hosts from the families Cannaceae, Costaceae, Marantaceae and Zingiberaceae. The family Heliconiaceae was removed from the sampling effort estimates, as the ITS2 barcode cannot be successfully amplified in plants of this family (Figure 3*B*). Using ITS2, it is possible to identify 99% of the host plants to genus after collecting ca. 400 rolled leaf beetles. Approximately 900 beetle samples are required to successfully identify 99% of the interactions to species (Figure 5*B*).

Intrinsic failure rates associated with DNA extraction, amplification and sequencing require that at least four samples of individuals feeding on a particular host plant be collected to identify a plant-herbivore interaction (probability of identifying the diet of collected herbivore = DNA extraction success × identification success per sequence, Table 1). Although diet identification using molecular markers requires a larger sample size than direct observations, an advantage of molecular methods is that they associate insect herbivores only with plants that were ingested. During field observations, researchers often assume that the plant where an insect was collected is its host, leading to potentially erroneous plant-herbivore associations.

Our sampling estimates were generated for this particular study system. In other plant-insect herbivore systems, the sampling effort needed to identify diets may be substantially higher than the one reported for rolled-leaf beetles. Because rolled-leaf beetles feed for several days on the same host plant, the cloning step required to identify multiple host plants in the same gut sample is avoided. However, in polyphagous herbivores that frequently move between host plants, Sanger sequencing of PCR amplicons might not be a practical alternative because of the high cost and effort involved in cloning.

One solution to determine the diets of polyphagous insect herbivores is DNA metabarcoding, i.e., the combination of DNA barcodes and next generation sequencing (NGS) technologies [20]. DNA metabarcoding can amplify thousands of sequences per PCR product and in theory it would be able of identifying several consumed species without cloning [18].

Although NGS has an enormous potential for the study of plant-herbivore interactions, this novel technique is affected by the same limitations that we described for traditional Sanger sequencing methods [21]. The accuracy of diet identification using NGS will also depend on the quality of sequences extracted from insect gut contents, the taxonomic coverage in the DNA barcode reference library, and the resolution of the molecular markers [21]–[23]. Therefore, researchers must be cautious and validate with complementary experiments each step in the process of diet identification.

### Conclusions

In conclusion, we tested in a community of rolled-leaf beetles the reliability of multiple DNA barcode loci for the identification of insect herbivore diets. DNA barcode techniques can identify insect host plants to the taxonomic level of family, genus and species. DNA barcodes are also successful at differentiating closely related host plant species allowing the successful reconstructing of multiple associations in plant-herbivore networks. As better DNA extraction methods are developed for degraded DNA sampled from insect guts and as more cost-effective and rapid next-generation DNA sequencing protocols are developed, we expect that reconstructing plant-herbivore networks with these methods will become the standard for a detailed understanding of these interactions.

## Materials and Methods

### Study Site and Species of Interest

Fieldwork was conducted from October 2008 to March 2011 at La Selva Biological Station, a tropical rain forest site in Costa Rica, Central America (10°26′N, 83°59′W). La Selva is classified as an aseasonal tropical wet forest [24], receiving an average of 4000 mm of rain per year [11]. Two mild dry seasons occur between January – April and September – October [25], [26].

This study focuses on insect herbivores in the genera *Cephaloleia* and *Chelobasis* (Coleoptera; Chrysomelidae) and Neotropical plants in the order Zingiberales (Families Cannaceae, Costaceae, Heliconiaceae, Marantaceae and Zingiberaceae) [27]–[29]. This interaction is one of the oldest and most conservative plant-herbivore associations [30], [31]. *Cephaloleia and Chelobasis* are also known as the “rolled-leaf beetles” because their lifecycle occurs inside the scrolls formed by the young rolled leaves of their host plants [13], [32]. Adult rolled-leaf beetles feed and oviposit on the young leaves of their host plants [12]. When the leaves unroll, adults fly to another young leaf [33], [34]. Young leaves of Zingiberales remain rolled for a few days to several months depending on the host plant species [35], [36]. Therefore rolled-leaf beetles usually feed on one host plant species for several days or weeks. This feeding behavior is very convenient for diet identification that combines molecular markers and Sanger sequencing, as gut contents will usually contain DNA from only one host plant. Therefore, DNA cloning is not needed to identify multiple hosts in each sample.

At La Selva 20 species of rolled-leaf beetles feed on 33 species of Zingiberales (Figure 2) [37]. The diet breadths of rolled-leaf beetles range from specialized species that feed on one host plant to generalists that feed on 17 host plants from three plant families (Figure 2) [37]. The alpha taxonomies of both rolled-leaf beetles and Zingiberales at La Selva have been thoroughly studied over the last 40 years [13], [37]–[43]. All necessary permits required for this field study were obtained from the Ministerio de Ambiente, Energía y Telecomunicaciones, Costa Rica (MINAE 164-2010-SINAC). Local research permission was granted by the Organization for Tropical Studies. This study did not involve endangered or protected species.

### Comprehensive Survey of Rolled-leaf Beetle – Zingiberales Interactions

During two years we surveyed the diets of all rolled-leaf beetles present in our study area. Surveys were performed during the rainy and dry seasons of each year. (First survey: October 2008–February 2009; Second survey: October 2010–February 2011). Surveys consisted in carefully unrolling each young leaf and counting the number of individuals of each rolled-leaf beetle species. The minimum distance between plants surveyed was 5 m (Total number of plants surveyed = 3092, Total number of rolled-leaf beetles recorded = 7359).

### DNA Barcode Libraries of Host Plants

We collected vouchers of at least three individuals of each species in the order Zingiberales at La Selva (Total of individuals = 141, Figure 2). DNA was extracted from 0.05 g of leaf tissue dried with Silica Gel. Plant vouchers were deposited in the United States National Herbarium, National Museum of Natural History, Smithsonian Institution (US).

Samples were transferred to 96 - well 2 ml Costar plates and capped. Tissue was disrupted by freezing with liquid nitrogen and grinded with zirconia beads using Qiagen Tissue Lyser. The disrupted tissue was incubated overnight in a GUSCN (guanadinium thiocyanate-tris HCl) buffer at 54°C. The lysate was then spun to pellet debris, and the cleared lysate mixed with GUSCN based binding buffer, then DNA was captured through binding on a 96 well glass-filter plate through centrifugation, washed twice and finally eluted in 200 µl of 10 µM Tris-HCl. All DNA extractions were conducted in a PCR free laboratory. Aliquots of DNA were prepared, with 30 µl material separated as a working stock for the PCR laboratory, and remaining DNA extract archived with 2-D barcode matrix tubes with numerical GUIDs (globally unique Identifier) used to track the samples throughout the process of PCR, sequencing [44]. DNA extracts are deposited in the National Museum of Natural History Biorepository - Smithsonian Institution.

### DNA Extractions from Insect Herbivore Gut Contents

To identify insect herbivore diets using DNA barcodes, we collected 1 to 8 rolled-leaf beetle individuals for each of the insect-host plant associations included in this study (total of insect herbivores = 244, total of insect-host plant associations = 74, Figure 2, Table S1). Rolled-leaf beetles were collected in the field, and after identifying the beetle and host plant species, each beetle was rinsed in water to remove any external contamination, then placed in a cryogenic tube and preserved in liquid nitrogen.

Two taxonomists independently identified each beetle species (C.L.S and C.G). High resolution 3D images were obtained for each specimen using an Auto-Montage® system (SYNCROSCOPY, Frederick, MD, USA). Images are available upon request to the corresponding author. Each beetle was placed into a separate screw-cap tub with an O-ring and DNA extraction was conducted in a PCR free laboratory, where no plant DNA extractions had been implemented. Each beetle sample had the thorax or entire body used in the extraction depending on the size of the individual. The remnant parts of each sample were deposited in the National Museum of Natural History Biorepository - Smithsonian Institution. A mixture of disposable silica and zircon beads (0.1–2.3 mm diameter) were added to each tube containing a beetle and disrupted with a single tube bead-mill.

We obtained high-quality DNA from insect gut contents by modifying DNA extraction techniques originally developed to obtain ancient DNA studies (Guanadinium-thyocyanate lysis buffer with glass fiber membrane recovery) [45]. Tubes with disrupted contents were opened one at a time and 300 µl of the plant based GUSCN extraction buffer was added [44]. Samples were mixed by vortexing then placed in a water bath at 54°C overnight. Following incubation, individual tubes were spun to pellet cellular debris. Each tube was open sequentially, and 100 µl of lysate transferred to individual tubes, mixed with binding buffer and then transferred to a 96-well plate for immobilization and isolation of DNA. Following mixture with lysis buffer, DNA extracts were treated similarly to plant DNA extractions, with DNA captured through binding to a glass-fiber filtration plate through centrifugation, washed twice following and then eluted with 200 µl of 10 µM Tris-HCl [44]. DNA extracts were archived in 2-D barcoded matrix tubes, each with a numerical GUID for post extraction tracking and processing. DNA extracts are deposited in the National Museum of Natural History Biorepository - Smithsonian Institution.

### DNA Sequencing Methods

To generate the reference plant DNA barcode library, three loci were sequenced for each of the vouchered samples: plastid *rbcL*, nuclear ribosomal ITS2 and plastid *trnH-psbA* intergenic spacer used in routine DNA barcoding [14], [15]. For ITS2 we used published primers (ITS2-2For and ITS3-Rev) that have been shown to preferentially amplify plant DNA and which have not been reported to exhibit high fungal recovery rates. PCR was conducted in 96-well plate formats using standard primers outlined in Table S2 [46]. PCR was followed by ExoSap purification of amplified products, and then subjected to standard sequencing using BigDye Di-Deoxy terminator sequencing. Recovered sequences were analyzed using Sequencher, with contigs constructed from forward and reverse sequences and exported as aligned FASTA files for construction of the host plant DNA reference library.

For the insect herbivore gut content samples, we attempted PCR recovery for each of the three loci in our reference database. For *rbcL*, we used a pair of mini-barcodes which divided the normal *rbcL* barcode region into two segments, one 230 bases long and a second 320 bases long. We used two internal primers that annealed to the same position to create the mini-barcode partitions in conjunction with the standard barcode primers. The rbcLa-230 was a reverse primer used with *rbcL*_F, and *rbcL* 260-F was used with the standard reverse DNA barcode primer rbcLa_Rev. The sequence of these *rbcL*-mini-barcodes is given in Table S2.

Similar to the use of the *rbcL* mini-barcode, we used primers for ITS2 that produced the smallest possible amplicon size, to increase the chance that we could recover intact sequences from potentially highly degraded plant DNA from insect gut contents. We used the same ITS2-2-FOR primer used in our reference library construction and the standard ITS4-Rev primer. ITS4_Rev produced a 360bp amplicon that was approximately 90bp shorter than the reference library sequences. We were unable to identify a primer that would generate a mini-barcode for the *trnH-psbA* region that would work for the entire order of Zingiberales, and thus we attempted recovery of forensic sequences with the full-length barcode region using standard primers.

PCR was conducted under standard conditions for each gene, with the exception that we increased the number of PCR cycles to 40 for all samples (*see* a summary of primers and PCR conditions in Table S2). All PCR were checked and then standard ExoSap and BigDye sequencing were applied. Following sequencing, we observed than many PCR products from the gut content samples failed to sequence cleanly. This result was primarily a problem for the ITS2 region. We subsequently took all PCR products for ITS2 that did not sequence readily, and subjected them to gel-isolation in TAE (Tris-Acetic Acid-EDTA) 2% low molecular weight resolving gels. Bands with PCR products were cut out of the gels, placed into individual tubes and purified with Qiagen Gel-extraction kits. The resulting purified products were used directly in Big-Dye sequencing reactions with standard primers.

### Identification of Insect Herbivore Diets Using Molecular Markers

DNA sequences from gut contents of rolled-leaf beetles were compared against the plant DNA barcode library generated by this project using the BLAST algorithm and default search parameters [47]. In cases when DNA sequences from gut contents matched multiple DNA sequences from different plant families, host plant identification was assigned to the order Zingiberales. In cases when top BLAST scores were equal for species from different genera within the same family, we identified such interaction to the family-level. If the best BLAST scores were equivalent for plant species within the same genus, interactions were assigned to the genus. We assigned diet identifications at the species-level only when the best scores matched reference sequences from the same plant species. Sequences from gut contents that did not match any of the sequences in the DNA barcode library were scored as not identified.

### Sampling Effort and Host Plant Identification Using DNA Barcodes

We estimated for the interactions included in this study the sampling effort required for reconstructing plant-herbivore associations using DNA barcodes. First we simulated random collections with replacement of 1000 rolled-leaf beetles from known host plants. For each collection event, we estimated the probability of successful host plant identification to a given taxonomic level using a given DNA barcode (i.e. family-, genus- or species-level) by multiplying the probability of success during DNA extractions and the probability of successful host plant identification to a given taxonomic level using a molecular marker (Table 1). We performed independent simulations for *rbcL* DNA sequences (for identifications to the family and genus taxonomic resolutions) and ITS2 DNA sequences (for identifications to the genus and species taxonomic resolutions). We did not perform this analysis for the *trnH-psbA* as we failed to amplify high-quality DNA from Zingiberales or insect gut contents (*see* results). Because insect collections were modeled at random, our sampling assumes equivalent occurrence for all plant-herbivore associations.

We generated accumulation models of plant-herbivore interactions using the Kindt’s exact accumulator (Equation 1) [26]. Where *Ŝ* represents the average number of plant-herbivore interactions. S_tot_ represents the total of interactions recorded in the survey.
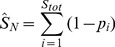
(1)


Where:
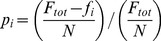
(2)


In our application of the Kindt’s exact accumulator, *N* indicates the numbers of collections for which the average number of interaction is calculated, *f_i_* represents the frequency of interaction *i*, and *F_tot_* is the total number of rolled-leaf beetles surveyed.

We estimated the variance for the accumulation curves:
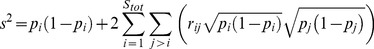
(3)


Where *r_ij_* is the correlation coefficient between interaction *i* and interaction *j*. Simulations and accumulation curves were estimated using Program R (package vegan) [48].

## Supporting Information

Table S1 **Data matrix to generate** **Figure 2** **.**
(DOCX)

Table S2 **Primers and PCR conditions used in this study to obtain DNA barcode libraries and sequences from insect gut contents.**
(DOCX)

Supplement S1 **DNA sequences (rbcL and ITS2) obtained from insect herbivore gut contents.**
(TXT)
