# Supplementary material for: Tropical Plant–Herbivore Networks: Reconstructing Species Interactions Using DNA Barcodes
Source: PLoS One. 2013 Jan 8;8(1):e52967. doi: 10.1371/journal.pone.0052967 (PMC3540088; doi:10.1371/journal.pone.0052967)
Supplement: Table S1 — Data matrix to generate Figure 2 . (DOCX) [file pone.0052967.s001.docx]

**SUPPORTING INFORMATION**

**TABLE S1.** Identification matrix for insect herbivore-host plant interactions in a tropical rain forest. This dataset generates Figure 3. Each cell represents an interaction between an insect herbivore species (rows) and a host plant species (columns). Host plants: **Heliconiaceae.** Him = *Heliconia imbricata*. Hir = *H. irrasa*. Hla = *H. latispatha*. Hmr = *H. mariae*. Hmt = *H. mathiasiae*. Hpo = *H. pogonantha*. Hwa = *H. wagneriana*. **Zingiberaceae.** Ral = *Renealmia alpinia*. Rce = *R. cernua*. Rpl = *R. pluriplicata*. **Costaceae.** Cbr = *Costus bracteatus*. Clae = *C. laevis*. Cmal = *C. malortieanus*. **Marantaceae.** Ccl = *C. cleistantha*. Ccr = *C. crotalifera*. Cgy = *C. gymnocarpa*. Cha = *C. hammelii*. Cin = *C. inocephala*. Clas = *C. lasiostachya.* Clu = *C. lutea*. Cma = *C. marantifolia*. Csi = *C. similis*. Cve = *C. venusta*. Cwa = *C. warscewiczii*. Iel = *Ischnosiphon elegans*. Iin = *I. inflatus*. Ppr = *Pleiostachya pruinosa*. **Cannaceae.** Ctu = *Canna tuerckheimii*.

**Matrix values:** NO INTERACTION (host plants not consumed by the insect herbivore species) = 0, NO MATCH (no match when DNA sequence is compared with the DNA barcode library) = 1. Identification to the: ORDER = 2, FAMILY =3, GENUS = 4, SPECIES = 5 taxonomic levels.
