# Supplementary material for: Tropical Plant–Herbivore Networks: Reconstructing Species Interactions Using DNA Barcodes
Source: PLoS One. 2013 Jan 8;8(1):e52967. doi: 10.1371/journal.pone.0052967 (PMC3540088; doi:10.1371/journal.pone.0052967)
Supplement: Table S2 — Primers and PCR conditions used in this study to obtain DNA barcode libraries and sequences from insect gut contents. (DOCX) [file pone.0052967.s002.docx]

**TABLE S2.** Primers and PCR conditions used in this study to obtain DNA barcode libraries and sequences from insect gut contents.

| PRIMER | SEQUENCE | ANNEALING TEMPERATURE (∘C) | NUMBER OF PCR CYCLES | REFERENCE |
| --- | --- | --- | --- | --- |
| ITS4_Rev | GCATATCAATAAGCGGAGGA | 49 | 40 | White et al. 1990 |
| ITS3_Rev | ATTGTAGTCTGGAGAAGCGTC | 49 | 40 | Chen et al. 2010 |
| ITS2-2_For | ATGCGATACTTGGTGTGAAT | 49 | 40 | Chen et al. 2010 |
| *rbcL*_F | ATGTCACCACAAACAGAGACTAAAGC | 55 | 35 | Soltis et al. 1992 |
| *rbcL*_Rev | GTAAAATCAAGTCCACCRCG | 55 | 35 | Kress et al. 2009 |
| *rbcL*_230_Rev | CTTACCAGYCTTGATCGTTACAAAGG | 50 | 40 | This publication |
| *rbcL*_260_For | CCTTTGTAACGATCAAGRCTGGTAAG | 50 | 40 | This publication |
| psbA | GTTATGCATGAACGTAATGCTC | 55 | 35 | Sang et al. 1997 |
| trnH | CGCGCATGGTGGATTCACAATCC | 55 | 35 | Tate & Simpson 2003 |

**White TJ, Bruns T, Lee S , Taylor J. 1990.** Amplification and direct sequencing of fungal ribosomal RNA genes for phylogenetics. In: Innis MA, Gelfand DH, Sninsky JJ and White TJ, eds. *PCR protocols: A guide to methods and applications*. New York: Academic Press. 315–322.

**Chen SL, Yao H, Han JP, Liu C, Song JY, Shi LC, Zhu YJ, Ma XY, Gao T, Pang XH, Luo K, Li Y, Li XW, Jia XC, Lin YL , Leon C. 2010.** Validation of the ITS2 region as a novel DNA Barcode for identifying medicinal plant species. *PLoS ONE* **5**.

**Soltis PS, Soltis DE , Smiley CJ. 1992.** An rbcL sequence from a Miocene *Taxodium* (bald cypress). *Proceedings of the National Academy of Sciences of the United States of America* **89:** 449-451.

**Kress WJ, Erickson DL, Jones FA, Swenson NG, Perez R, Sanjurb O , Bermingham E. 2009.** Plant DNA barcodes and a community phylogeny of a tropical forest dynamics plot in Panama. *Proceedings of the National Academy of Sciences of the United States of America* **106:** 18621-18626.

**Sang T, Crawford DJ , Stuessy TF. 1997.** Chloroplast DNA phylogeny, reticulate evolution and biogeography of *Paeonia* (Paeoniaceae). *American Journal of Botany* **84:** 1120-1136.

**Tate JA , Simpson BB. 2003.** Paraphyly of *Tarasa* (Malvaceae) and diverse origins of the polyploid species. *Systematic Botany* **28:** 723-737.
